# Double burden of malnutrition among women of reproductive age: Trends and determinants over the last 15 years in India

**DOI:** 10.1371/journal.pone.0304776

**Published:** 2024-06-13

**Authors:** Ivan James Prithishkumar, Marimuthu Sappani, Varsha Ranjan, Chhavi Garg, Thenmozhi Mani, Malavika Babu, Melvin Joy, Bhawna Rao, Edwin Sam Asirvatham, Jeyaseelan Lakshmanan

**Affiliations:** 1 Mohammed Bin Rashid University of Medicine and Health Sciences, Dubai Healthcare City, Dubai, UAE; 2 Department of Biostatistics, Christian Medical College, Vellore, Tamil Nadu, India; 3 Independent Public Health Consultant, New Delhi, India; 4 Faculty of Science, Global Health: Research, Vrije Universiteit, Amsterdam, Netherlands; 5 Centre for Trials Research, College of Biomedical and Life Sciences, Cardiff University, Cardiff, United Kingdom; 6 Biostatistician, Translational and Clinical Research Institute, Faculty of Medical Sciences, Newcastle University, Newcastle upon Tyne, England, United Kingdom; 7 National AIDS Control Organization (NACO), New Delhi, India; 8 Health Systems Research India Initiative (HSRII), Trivandrum, Kerala, India; University of Botswana, BOTSWANA

## Abstract

**Introduction:**

Double burden of malnutrition (DBM) has been recognized by the World Health Organisation (WHO) as an emerging Global Syndemic characterized by the simultaneous occurrence of both undernutrition and overnutrition. Women of the reproductive age group (15 to 49 years) are disproportionately affected by DBM and are at high risk of continuing the intergenerational cycle of malnutrition. This study aims to assess the changing trends and determinants of DBM among women of the reproductive age group in India.

**Materials and methods:**

We used data from three rounds of National Family Health Surveys (NFHS-3,4,5) conducted in years 2005–06, 2015–16, and 2019–2021. Descriptive statistics and Poisson regression analysis were done using weights with log link function.

**Results:**

The prevalence of anaemia, underweight and overweight/obesity was 57.2%, 18.6% and 24% respectively. The combined burden of underweight and anaemia has declined by 46% (21.6% to 11.7%), whereas the combined burden of overweight/obesity and anaemia has increased by 130% (5.4% to 12.4%) in the past 15 years. The prevalence of DBM, which includes both underweight and overweight/obesity with anaemia was 24.1% in 2021, a decline of 11% in 15 years. Women who were younger, rural, less educated, poor and middle class, and women living in the eastern, western and southern regions of India had higher risk for being underweight with anaemia and lower risk for developing overweight/obesity with anaemia.

**Conclusion:**

The significant decrease in underweight yet enormous increase in overweight/obesity over the past 15 years with the persistence of anaemia in both ends of the nutritional spectrum is characteristic of the new nutritional reality emphasizing the need to address malnutrition in all its forms. It is critical to consider geography and a population specific, double-duty targeted intervention to holistically address the risk factors associated with DBM and accomplish India’s commitment to the global agenda of Sustainable Development Goals-2030.

## Introduction

Double burden of malnutrition (DBM) has been recognized by the World Health Organisation (WHO) as an emerging Global Syndemic characterized by the simultaneous occurrence of both undernutrition and overnutrition [1–4]. Although dietary excess, obesity, and overnutrition have historically been considered as separate entities from undernutrition and nutritional deficiencies, there is increasing evidence that both exist together and can be seen at the level of the individual [4–10]. DBM at the population level is defined as the co-existence of overweight/obesity with deficiency of vitamins or minerals and nutritional anaemia, and underweight/stunting with similar nutritional deficiencies; DBM at the individual level can occur when the same individual can be both stunted and overweight [2, 11, 12]. Researchers including the WHO emphasize that these two entities should no longer be addressed in silos, but with a simultaneous double-duty targeted approach as this new nutritional reality of DBM affects almost every low and middle-income country (LMIC) [2–4, 13].

As per the Global Nutrition Report 2018, most governments worldwide are struggling to deal with the new public health reality of interconnected undernutrition and overnutrition, which disproportionately affects women, children, and the poor, with enormous human and economic costs [4, 8, 14, 15]. India continues to be the largest contributor to the global prevalence of malnutrition with the largest share of the world’s undernourished population [16]. On the other hand, the country experiences a gradual increase in overweight/obesity [17, 18]. As per the National Family Health Survey—4 (NFHS) conducted in 2015–16, the prevalence of both underweight and overweight was around 41% among women. Specifically, women of the reproductive age group (aged 15–49 years) are disproportionately affected by underweight (23% of women and 20% of men), and overweight/obese (21% of women and 19% of men) [19]. Malnutrition among them, specifically nutritional deficiencies during pregnancy, leads to a higher occurrence of foetal malnutrition and infant mortality, pregnancy-related complications resulting in increased maternal morbidity and mortality accounting for about three million global deaths annually [19–21].

Over the last decade, India has ardently attempted to address undernutrition through several programmes but with limited success. Though the burden of undernourishment and malnutrition has decreased due to governmental interventions, increased agricultural productivity and economic growth, extreme hunger and malnutrition combined with the increasingly obesogenic environment are escalating the evolving burden of DBM in India [17, 22–24]. Malnutrition in women of the reproductive age group has a significant consequence in two critical windows—periconception and the early neonatal period (5). LMIC’s have experienced intergenerational cycles of severe maternal malnutrition associated with increased risk of micronutrient deficiency and stunting in childhood. Similarly, maternal obesity when combined with gestational diabetes can result in increased foetal adiposity and insulin resistance in subsequent generations [5]. Thus, women of the reproductive age group are key risk groups that are responsible for continuing the DBM paradox by generating malnourished birth outcomes [19]. This intergenerational emergence of DBM is seen to span across generations escalating the growing global crisis [5].

The dual burden of underweight and overweight/obesity with associated anaemia and other nutritional deficiencies among the population of India remains yet to be fully explored. While it is essential to understand the extent of the problem and trends over time, it is imperative to understand the determinants of the new nutrition reality especially among women of the reproductive age to develop and implement evidence-based programmes and policies towards achieving the Sustainable Development Goals (SDG).

This research aims to assess the trends and determinants of the changing patterns of DBM with anaemia among non-pregnant women of the reproductive age group from 2005 to 2021 in India. The study results will be interpreted in the context of social and economic development, behavioural patterns of the population, and national programmes to address the challenges of DBM in India.

## Materials and methods

We used three rounds of the large-scale National Family Health Surveys (NFHS-3, 4 and 5), conducted on a representative sample of households across the country in 2005–06, 2015–16 and 2019–2021. NFHS is a multi-round survey performed in a representative sample of households across India that provides information on various indicators including maternal and child health (MCH) in India. The survey employed a stratified two-stage sampling method, and each district was stratified into rural and urban areas according to the place of residence. We abstracted the open-source women (Individual Recode) data from the Demographic and Health Survey (DHS) https://dhsprogram.com/data/dataset_admin. These cross-sectional surveys collected detailed information on population, health, and nutrition.

### Independent variables

The socioeconomic, demographic, clinical and behavioural covariates included in the analysis were age, education, occupation, wealth and wealth index, anaemia, parity, and zone. Age was categorised into four groups such as 15–19, 20–29, 30–39, and 40–49 years. Education was categorized as no education, primary, secondary, and higher education. Occupation of the respondent was classified as employed and unemployed.

For the wealth index, the poorest and poor were combined as “POOR” category and the rich and richest were combined as “RICH”, but the middle-income category was retained as the same. Anaemia was categorized as non-anaemic, and any form of anaemia–mild, moderate and severe. Among non-pregnant women, the cut-off Haemoglobin (Hg) value of anaemia was <12.0 g/dl. Parity, defined as the number of children ever born, was categorised as 0, 1, 2, 3 or more. The states were grouped into five zones that are north, east, west, south and northeast.

### Dependent variables

The double burden of malnutrition was the outcome variable and was based on the Body Mass Index (BMI) and Haemoglobin (Hb) level of non-pregnant women of reproductive age group. As recommended by WHO, BMI is categorized as underweight—<18.5 kg/m^2^; normal—18.5 to 24.9 kg/m^2^; and overweight/obesity - ≥ 25.0 kg/m^2^. Women having a Hb level of <12 g/dl were considered anaemic. Presence of both (i) underweight and anaemia, and (ii) overweight/obesity and anaemia, within the same population group was categorised as having DBM. Women who were exclusively having anaemia, normal weight, overweight/obesity and underweight were not included in the DBM category.

### Statistical analysis

The data analysis was done using SPSS 25 and STATAIC 16.0. Descriptive statistics and adjusted analyses (adjusted for all covariates) were done. To account for the two-stage complex survey design of the study, the key survey elements stratum and clusters were used to calculate weights. All the analyses were done using ‘survey weights’ given in the dataset. We have followed the procedure given by the DHS for analysis using the “survey” module STATA command, which is adjusted for cluster and strata as well. First, the survey utilities were set up using “svyset” command then the regression model was fitted using “svy linearized” command [25]. The Poisson regression analysis was done using weights with log link function as logistic regression with log link is not converged. Therefore, the risk ratios with 95% CI are presented. P value <0.05 was considered as the level of significance.

### Ethical considerations

The analysis was approved by the Institutional Review Board of Christian Medical College (CMC), Vellore, India. The study is based on secondary data analysis with the dataset available in the public domain for open use.

## Results

### Prevalence of anaemia, underweight, overweight/obesity and DBM

The prevalence and its trends of anaemia, underweight, overweight/obesity and DBM among non-pregnant women of the reproductive age group (15–49 years) over the last 15 years are presented in Table 1. In 2021, the prevalence of anaemia was 57%, an increase of 4% from 2006. While the prevalence of underweight decreased by 48%, overweight/obesity increased by 90% in 15 years. The combined burden of underweight and anaemia declined by 46% (21.6% to 11.7%), whereas the combined burden of overweight/obesity and anaemia increased by 130% (5.4% to 12.4%) in 15 years. The prevalence of DBM in 2021, which includes both underweight and overweight/obesity with anaemia was 24.1%, a decline of 11% in 15 years.

**Table 1:** Prevalence of DBM among non-pregnant women by NFHS surveys 3, 4 and 5.

	NFHS 3 (117956)		NFHS 4 (668563)		NFHS 5 (696990)	
	n	%	n	%	N	%
**Anaemia**						
Not Anaemic	49706	44.9	303710	46.8	280710	42.8
Anaemic	61121	55.1	345409	53.2	375521	57.2
**BMI**						
Underweight (≤18.5)	40116	35.5	149067	22.9	124182	18.6
Normal (18.5 to 24.99)	58687	51.9	368834	56.5	382292	57.4
Overweight & Obese (≥25)	14226	12.6	134378	20.6	159607	24.0
**Double Burden of Malnutrition**						
Not Anaemic & Normal BMI	26255	23.7	171455	26.5	159831	24.4
Anaemic & Normal BMI	31142	28.1	194907	30.1	216701	33.1
Not Anaemic & Underweight	15509	14.0	60977	9.4	45549	7.0
Not Anaemic & Overweight /Obese	7863	7.1	70650	10.9	74701	11.4
Anaemic and Underweight	23856	21.6	87190	13.5	76870	11.7
Anaemic and Overweight /Obese	6013	5.4	62518	9.7	80931	12.4
**Double burden**						
No	80768	73.0	497989	76.9	496782	75.9
Yes **(Underweight and Overweight/Obesity with Anaemia**)	29869	27.0	149708	23.2	157801	24.1

### Prevalence of DBM by socio-economic, and demographic characteristics

As described in Table 2, DBM has been consistently high in the eastern region of India during the last 15 years accounting for more than a quarter of the burden, while the northeast region consistently demonstrated a lower burden of DBM during the same period. The rural areas recorded a 15% reduction of DBM over the past 15 years, while the urban areas remained at the same level. The DBM is relatively higher among the adolescent (15–19 years) and elderly age group (40–49 years) across all survey periods. The prevalence of DBM among women without formal education was relatively higher in 2006 and 2016, however, it was higher among women with primary and secondary education in 2021. DBM among the poor declined by 27% whereas it increased by 9% among rich women. DBM decreased with an increase in the number of children.

**Table 2:** Weighted prevalence of DBM among non-pregnant women by socio-demographic factors.

	Double Burden of Malnutrition								
	NFHS 3 (2005–06)			NFHS 4 (2015–16)			NFHS 5 (2019–21)		
	Total	n	%	Total	n	%	Total	n	%
**Overall**	110637	29869	27.0	647697	149708	23.10	654583	157809	24.10
**Zone**									
North	30622	6891	22.5	170534	36868	21.6	198748	42330	21.3
East	25021	8257	33.0	142844	36365	25.5	150723	40074	26.6
Northeast	4274	1144	26.8	22816	3780	16.6	24681	5010	20.3
West	25556	6908	27.0	149526	34386	23.0	145296	35988	24.8
South	25164	6668	26.5	147522	35064	23.8	135136	34400	25.5
**Age**									
15–19	21879	6101	27.9	113623	28770	25.3	111212	29974	27.0
20–29	36398	9564	26.3	208017	44184	21.2	203999	44172	21.7
30–39	30957	8259	26.7	178272	40043	22.5	182999	43758	23.9
40–49	21403	5946	27.8	147785	36711	24.8	156373	39897	25.5
**Residence**									
Urban	35472	8766	24.7	221629	52377	23.6	207785	51462	24.8
Rural	75165	21103	28.1	426068	97331	22.8	446798	106339	23.8
**Education**									
No education	44588	13379	30.0	179682	42271	23.5	149206	34128	22.9
Primary	16543	4481	27.1	81340	19226	23.6	78055	18971	24.3
Secondary	41697	10457	25.1	306023	72090	23.6	328565	83465	25.4
Higher	7801	1549	19.9	80653	16121	20.0	98757	21238	21.5
**Wealth**									
Poor	40036	13117	32.8	241958	57603	23.8	254568	60733	23.9
Middle	22657	5883	26.0	133876	29655	22.2	136384	32009	23.5
Rich	47944	10869	22.7	271863	62450	23.0	263629	65058	24.7
**Occupation**									
Unemployed	62295	16017	25.7	76745	18109	23.6	67239	16010	23.8
Employed	48301	13843	28.7	33288	7890	23.7	30427	7213	23.7
**Parity**									
No Children	1428	428	30.0	33923	8394	24.7	29819	7737	25.9
Single Child	9600	2338	24.4	47867	10997	23.0	48087	12031	25.0
2 Children	66100	17548	26.5	411506	95017	23.1	438734	107321	24.5
3+ Children	30729	8778	28.6	150453	34353	22.8	132410	29267	22.1

### Determinants of DBM among non-pregnant women of reproductive age group

The results of the multivariable analysis that was carried out separately for both the sub-categories of DBM is presented in Table 3.

**Table 3:** Multivariable analysis for DBM among non-pregnant women.

Variables	Underweight and Anaemia		Overweight/Obesity and Anaemia	
	RR (95% CI)	P value	RR (95% CI)	P value
**Year of Survey**				
NFHS 3 (2005–06)	Ref		Ref	
NFHS 4 (2015–16)	0.71 (0.69,0.73)	<0.001	1.44 (1.38, 1.50)	<0.001
NFHS 5 (2019–21)	0.66 (0.64,0.67)	<0.001	1.77 (1.70, 1.85)	<0.001
**Age**				
15–19	2.3 (2.25,2.35)	<0.001	0.19 (0.18, 0.20)	<0.001
20–29	1.59 (1.56,1.62)	<0.001	0.48 (0.46, 0.49)	<0.001
30–39	1.1 (1.08,1.13)	<0.001	0.84 (0.82, 0.85)	<0.001
40–49	Ref		Ref	
**Residence**				
Urban	Ref		Ref	
Rural	1.12 (1.09,1.14)	<0.001	0.83 (0.81, 0.85)	<0.001
**Wealth**				
Poor	1.53 (1.5,1.57)	<0.001	0.51 (0.50, 0.53)	<0.001
Middle	1.25 (1.22,1.28)	<0.001	0.77 (0.75, 0.78)	<0.001
Rich	ref		Ref	
**Education**				
No education	1.45 (1.41,1.49)	<0.001	0.93 (0.90, 0.96)	<0.001
Primary	1.22 (1.18,1.26)	<0.001	1.11 (1.08, 1.15)	<0.001
Secondary	1.17 (1.14,1.2)	<0.001	1.15 (1.12, 1.18)	<0.001
Higher	Ref		Ref	
**Zone**				
North	Ref		Ref	
East	1.25 (1.22,1.28)	<0.001	1.21 (1.18, 1.24)	<0.001
Northeast	0.91 (0.88,0.94)	<0.001	0.85 (0.82, 0.88)	<0.001
West	1.34 (1.31,1.37)	<0.001	0.88 (0.86, 0.91)	<0.001
South	1.11 (1.09,1.14)	<0.001	1.21 (1.18, 1.24)	<0.001
**Parity**				
No Children	Ref		Ref	
Single Child	0.93 (0.9,0.96)	<0.001	1.04 (0.99, 1.09)	0.124
2 Children	0.9 (0.88,0.93)	<0.001	0.99 (0.96, 1.03)	0.847
3+ Children	0.89 (0.87,0.92)	<0.001	0.92 (0.88, 0.95)	<0.001

**Note: Outcome DBM**: We defined DBM as, the presence of both (i) underweight and anaemia, and (ii) overweight/obesity and anaemia.

**Covariates:** Year of survey, age, residence, wealth, education, geographical zone and parity.

After adjusting for covariates, the risk of being underweight and anaemia declined by 29% in 2015 and 34% in 2021 compared to the year 2006 (p < .001) and it declined with the reduction in age. Rural women had 1.12 times higher risk compared to urban women; middle-class and poor women had 1.25- and 1.53-times higher risk for being underweight and anaemia. Similarly, women with less education had a higher risk for being underweight and anaemia. Women with one or more children had less risk for underweight and anaemia compared to mothers with no children (p < .001). The eastern, western and southern regions had higher risk for underweight and anaemia, compared to the northern region (p<0.001).

On the other hand, after adjusting for covariates, the risk of being overweight/obesity and anaemia increased by 44% in 2015 and 77% in 2021 compared to year 2006 (p < .001). Younger, rural, poor or middle class, less educated and women with 3 or more children had significantly lower risk for overweight/obesity and anaemia compared to other groups (p < .001). As compared to the northern region, the southern and eastern regions had 21% higher risk; the northeastern and western regions had 15% and 12% lower risk for overweight/obesity and anaemia respectively (p<0.001).

## Discussion

This research which is based on three large-scale national-level surveys across India is unique and describes the burden of DBM, one of the emerging and major public health issues, its trends over the last two decades and the associated factors. The key finding of programmatic importance is the juxtaposed shift in the trends of the two nutritional indicators, and the new nutritional reality of the coexistence of undernutrition and overweight/obesity, not only in India but in several countries across the world [26]. While the prevalence of overweight/obesity with anaemia indicates an increasing trend, there is an apparent decline of underweight and anaemia among non-pregnant women of reproductive age group which is in corroboration with existing literature across the world [17, 19, 27–30].

The prevalence of overweight/obesity and anaemia among women of reproductive age group was 12.4% in 2019–21 and the increasing prevalence over the last two decades, especially the higher risk among urban, wealthy and educated women is indeed a concern. However, this is not surprising and it is in alignment with published literature indicating a higher prevalence of overweight/obesity and anaemia among the socio-economically advantaged and in urban areas [16, 19, 31, 32]. As observed in our study, the higher risk for overweight/obesity and anaemia among older women is consistent with similar studies conducted in different parts of the world [16, 26]. This could be due to the associated lifestyle and behavioural factors in this age group leading to a shift in dietary patterns coupled with increased availability and access to energy-dense, processed foods, sugary beverages, and unhealthy snacks [19]. The high prevalence of overweight/obesity and anaemia is a critical public health issue as obesity is identified as a major risk factor and primary reason for the rapid increase in non-communicable diseases in India [16, 33, 34].

The study indicated a significant reduction in the prevalence of underweight and anaemia in the last two decades which is in alignment with the global trends. Globally, the age-standardised mean BMI increased from 22.6 kg/m^2^ in 1985 to 24.7 kg/m^2^ in 2017 in women [35]. During this time, the age-standardised global prevalence of underweight decreased from 14.6% to 9.7% in women. At the same time, the prevalence of anaemia increased slightly in India in comparison with the global trends that indicated a slight decline from 31% in 2000 to 30% in 2019 [36]. This declining trend in underweight in India could be due to the policy and programmatic efforts of various stakeholders that predominantly focused on undernutrition and anaemia besides, the notable improvement in the socio-economic status of the country [37]. In contrast to overweight/obesity and anaemia, higher age, rural, poor and less educated had greater risk for underweight and anaemia. A systematic review and meta-analysis in southeast Asia indicated a higher prevalence of underweight in rural areas compared to urban areas, and a higher prevalence of overweight in urban areas than in rural areas which co-relates with our study [29, 30]. In line with the present study, several studies have highlighted higher age, poor women and women with less education as risk factors for undernutrition and anaemia [30, 38, 39]. Geographically, compared to the northern region of India, eastern, western, and southern zonal regions had higher risk for underweight and anaemia which is contrary to earlier reports where southern states such as Kerala and Tamil Nadu were documented as “positive deviants” in some nutrition-related studies compared to northern states [40].

One fourth of Indian women of reproductive age (24%) are either underweight or overweight/obesity with anaemia. The juxtaposed trends of underweight and overweight, the remarkable decrease in underweight and the sharp increase in overweight/obesity have been documented across the world especially in several LMIC’s [29, 41]. However, the persistence of nutritional anaemia in both ends of the malnutrition spectrum over the 15-year period increases the vulnerability and intergenerational repercussions of DBM in India. There are many causes postulated for the co-occurrence of under- and overnutrition with additional deficiencies of micronutrients such as iron, zinc, iodine, vitamin A or D [7, 8]. Prolonged childhood malnutrition can result in metabolic dysregulation, altered signalling of insulin, and dysbiosis of the normal gut microbiome [6]. When such individuals with a stunted capacity for homeostasis are exposed to high metabolic foods in the course of life, it may result in overweight/obesity (OWOB) with a persistence of nutritional deficiency [5, 7, 8]. DBM in LMICs is commonly attributable to the global nutrition transition where there is easy availability of sub-optimal diets consisting of ultra-processed, cheap food and beverages which have high calorific yet significantly low nutritive value [2, 5, 6, 42]. There is also a significant generational change in lifestyle such as decreased physical activity, addiction to technology and increased leisure. This may result in an increased incidence of overweight/obesity in a previously undernourished individual with a persistent micronutrient deficiency and anaemia leading to the classic occurrence of DBM [9, 43]. Other factors such as gender, household size, socio-economic status, smoking, co-morbidities such as diabetes and hypertension, and geo-political factors such as war, global trade and food production are also key factors contributing to DBM [6, 7, 9]. The occurrence of a global pandemic such as COVID, SARS etc. can also precipitate the burden of DBM not only in India, but across the world [6, 44, 45].

Moreover, India’s nutritional programmes are largely targeted to improve undernutrition and anaemia in the population which may not be adequate to comprehensively address the substantial burden of DBM. The current pattern and high burden of DBM especially the overweight/obesity and anaemia challenges the effectiveness of the existing nutritional programmes in holistically addressing India’s commitment to the global agenda of Sustainable Development Goal 2 (SDG-2) that stresses on eradicating all forms of malnutrition including anaemia, specifically achieving the goal 2 “Zero hunger” and goal 3 “Good health and well-being”. India’s national initiatives especially Poshan Abhiyaan have indeed resulted in better outcomes in terms of reducing stunting and underweight among women of the reproductive age group [46]. Further, it is encouraging that India has launched the National Nutrition Mission (NNM), with ambitious NNM targets to reduce malnutrition. However, the current situation and trends indicate the need for substantial efforts to close the nutritional gap and achieve the national targets as well as the SDG-2030 goals.

### Limitations

Being a cross-sectional study, the cause-and-effect relationship could not be established. For instance, there is a strong association between socioeconomic status and DBM, which could be bi-directional. Though systems are calibrated against standard tools, the upgraded model of the analyser used to measure Hb in subsequent surveys could have affected the Hb measurements during the different surveys. Due to the large number of subjects studied, even a small difference or risk turns out to be statistically significant.

## Conclusion

The decrease in underweight and the significant increase in overweight or obesity in the population, with persistence of nutritional anaemia over the past 15 years indicate the transformation in dietary habits and behaviours of women resulting in noteworthy shifts in the nutritional trends in India. This study provides comprehensive evidence to understand the new nutritional reality emphasizing the need to address malnutrition in all its forms by envisioning and adopting new approaches and redesigning programmes and policies to bring about a significant improvement in the nutritional status of the masses across the nations. It is critical to have geography and population-specific targeted interventions addressing the specific risk factors of different categories of DBM. Evidence-driven policies and strategies involving true collaboration between communities, governments and other stakeholders are imperative to address the current nutrition realities and bring about real changes in the quality of life, and better health among the huge population of women of reproductive age group in India.
